# Hygiene along the continuum of care in the early post-natal period: an observational study in Nigeria

**DOI:** 10.1186/s12884-020-03282-3

**Published:** 2020-10-06

**Authors:** Yolisa Nalule, Helen Buxton, Erin Flynn, Olutunde Oluyinka, Stephen Sara, Oliver Cumming, Robert Dreibelbis

**Affiliations:** 1 Disease Control Department, London School of Hygiene and Tropical Medicine, London, WC1E 7HT UK; 2 Infection and Immunity, South Australian Health and Medical Research Institute, Adelaide, 5000 Australia; 3 Maternal and Child Survival Program (MCSP)/Save the Children Nigeria, Abuja, Nigeria; 4 Maternal and Child Survival Program (MCSP)/Save the Children US, Washington, DC 20036 USA

**Keywords:** Hand hygiene, Healthcare associated infections, Post-natal, Neonatal infection, Infection prevention and control, Newborn care, Nigeria, Maternal infection

## Abstract

**Background:**

Newborns delivered in healthcare facilities in low- and middle-income countries are at an increased risk of healthcare associated infections. Facility–based studies have focused primarily on healthcare worker behaviour during labour & delivery with limited attention to hygiene practices in post-natal care areas and largely ignore the wide variety of actors involved in maternal and neonatal care.

**Methods:**

This exploratory mixed-methods study took place in six healthcare facilities in Nigeria where 31 structured observations were completed during post-natal care, discharge, and the first 6 hours after return to the home. Frequency of hand hygiene opportunities and hand hygiene actions were assessed for types of patient care (maternal and newborn care) and the role individuals played in caregiving (healthcare workers, cleaners, non-maternal caregivers). Qualitative interviews with mothers were completed approximately 1 week after facility discharge.

**Results:**

Maternal and newborn care were performed by a range of actors including healthcare workers, mothers, cleaners and non-maternal caregivers. Of 291 hand hygiene opportunities observed at health facilities, and 459 observed in home environments, adequate hand hygiene actions were observed during only 1% of all hand hygiene opportunities. Adequate hand hygiene prior to cord contact was observed in only 6% (1/17) of cord contact related hand hygiene opportunities at healthcare facilities and 7% (2/29) in households. Discharge advice was infrequent and not standardised and could not be remembered by the mother after a week. Mothers reported discomfort around telling non-maternal caregivers to practice adequate hand hygiene for their newborn.

**Conclusions:**

In this setting, hand hygiene practices during post-natal care and the first 6 hours in the home environment were consistently inadequate. Effective strategies are needed to promote safe hand hygiene practices within the post-natal care ward and home in low resource, high-burden settings. Such strategies need to target not just mothers and healthcare workers but also other caregivers.

## Background

In populations with a high burden of neonatal mortality, up to half of all neonatal deaths are caused by infections, many of which are transmitted at the time of childbirth [1, 2]. Facility-based births are essential to providing safe, quality healthcare to mothers and newborns at the time of childbirth. However, newborns born in healthcare facilities (HCF) in low- and middle-income countries (LMIC) are at an increased risk of early onset sepsis due to unhygienic care practices during childbirth and post-natal care [3, 4]. Estimates suggest that newborns delivered in HCF in LMIC have 3–20 times greater risk of healthcare associated infections (HCAI) compared to newborns delivered in facilities in high income countries [3, 5]. However, this gap is likely to widen, with increasing proportions of women in LMIC giving birth at HCFs lacking robust infection prevention and control and hygiene management practices [1, 5–8].

Hand hygiene practices are an essential component of infection prevention and control (IPC) strategies in newborn and maternal care [9–11]. Improved handwashing practices by birth attendants and mothers have been associated with a 19% and 44% reduction in neonatal mortality, respectively [9]. To promote adequate hand hygiene in healthcare settings globally, the World Health Organisation (WHO) has published hand hygiene guidelines for healthcare workers (HCW) on key moments for hand hygiene during patient care [12–14]. Hand hygiene is also included as part of WHO recommended essential practices during newborn care - specifically before and after handling the newborn, before and after cord care, and after diaper changing [15].

Many HCF-based studies on hand hygiene practices during maternal and newborn care in LMIC have focused on HCW hand hygiene during labour and delivery or high-risk environments such as the neonatal intensive care units [16–20]. Community-based studies have generally focused on caregiver hygiene practices for newborns who are born outside the health facility or during the late post-natal period (> 7 days after birth) [18, 21–25]. Limited attention has been given to understanding hand hygiene compliance by the wide range of personnel and caregivers providing facility- and home-based newborn care in the immediate and early post-natal period (< 8 days after birth) [26, 27]. Understanding hand hygiene practices of various caregivers during this early post-natal period and how these are influenced by multiple factors allows for the design and implementation of more effective facility-based interventions to improve behaviours. The objective of this study is to document observed hand hygiene practices during the early post-natal care period in the healthcare facility and the first 6 hours after returning home.

## Methods

### Study design

The data presented here were collected over 4 weeks in July 2017 as part of a larger mixed-methods study investigating hygiene practices during childbirth, post-natal care, and return to the home environment across six health facilities in Kogi and Ebonyi states. Data reported here focus on the post-natal care ward, facility discharge, and the home environment. Findings related to IPC standards and infrastructure and practices during labour and delivery have been reported elsewhere [28, 29].

Facilities sampled for this study were all participating in the Maternal and Child Survival Program (MCSP) quality of care improvement program funded by the United States Agency for International Development (USAID). While all participating facilities received interventions aimed at improving the quality and utilisation of maternal and neonatal healthcare services, focus on hygiene was very limited. Further details of the MCSP quality of care improvement programme and facility selection are described in previous publications [28, 29]. In both Kogi and Ebonyi states, we sampled one facility at each of three levels: one primary HCF, a secondary HCF, and a tertiary HCF.

### Data collection

Facility-level data collection has been previously described [28, 29]. In brief, a structured facility observational checklist and a needs assessment survey were completed on the first day of observation in each participating HCF.

#### Structured observations

Structured observations were completed during childbirth, post-natal care, discharge, and the first 6 hours after returning home. A total of 39 women were recruited across the six facilities for at least one observation period. As a descriptive exploratory study, sample size was based primarily on resources availability. Participant eligibility criteria and observations of hand hygiene during childbirth have been previously reported [28]. Post-natal care observations began when the mother and baby were moved from the delivery area to the maternity/post-natal ward, hereafter referred to as post-natal care ward. Data collection staff documented observed hygiene practices and actions of all individuals involved in any maternal and newborn care for a period of up to four continuous hours or until the mother was discharged.

At the time of discharge, data collection staff directly observed and documented the discharge process with emphasis on recording discharge instructions the new mother received, particularly any reference to hygiene, handwashing and cord care. After discharge, staff accompanied the woman and newborn to her home to observe the hand hygiene practices there. Home observations lasted from the time of arrival in the home for up to 6 hours. At the home, key observations included newborn care practices (breastfeeding, bathing, diaper changes and handling), hand hygiene practices of all individuals involved in any newborn care, and other standard hand hygiene opportunities (feeding of self or others, visiting toilet, food preparation). At the end of the home observation, a structured spot check was completed of the home environment to assess the presence and availability of water, sanitation, and hygiene facilities in the home.

#### Semi structured interviews

The questionnaire used, data collection methods and analysis for these interviews have been previously published [29]. Semi-structured interviews were completed with one facility head, nurse or midwife, cleaner and mother per participating facility for a total of 18 HCF staff and 6 mothers. Mothers who consented to a second home visit had their phone numbers recorded at the end of the home observation. On the fifth day following the home visit, a selected mother was called to arrange the 45-min interview, which was then conducted in person at her home, at least 7 days after the initial visit. All household interviews were audio recorded and conducted in Yoruba, Igbo or English in two teams of two female local enumerators (an interviewer and a note taker), with prior experience of conducting qualitative research.

### Data analysis

All quantitative data were analysed using StataSE 15 (Stata Corp, College Station, TX, USA). Data from the facility needs assessment and walk through tools were examined descriptively to provide context for structured observation findings. Qualitative notes recorded during the observation data collection were reviewed and where applicable, recoded using STATA.

Observation data analysis was descriptive and focused on frequency and sequence of hand hygiene opportunities and associated hand hygiene actions based on WHO’s Five Moments for Hand hygiene and the three moments adapted for community neonatal hand hygiene [30, 31]. For the purposes of analysis, hand hygiene “opportunities” were defined as any activity that put hands at potential risk of contamination or activities that resulted in possible transmission of infectious agents to the mother and/or newborn during the observed period. Hand hygiene opportunities related to maternal care included: conducting clinical procedures on mothers (intramuscular injections, intravenous (IV) procedures), changing of perineal pads and emptying urine pots. Hand hygiene opportunities related to newborn care included direct cord contact via cord cleaning or cord inspection as well as activities during newborn care that could result in unobserved cord contact such as changing nappies, changing the newborn’s clothes, and skin contact with the newborn’s body. Hand hygiene “actions” were defined as any action taken in response, proactively or reactively, to a hygiene opportunity in an effort to mitigate potential infection transmission. Observed hand hygiene actions associated with each hand hygiene opportunity were coded into three categories in analysis. First, *no action* or *action* was assigned to any hand hygiene opportunity when there was no observed hand hygiene action taken or action taken. Hand hygiene actions were further coded as *adequate* (handwashing with soap and water) or *inadequate* (wearing gloves without handwashing with soap or rinsing with water only).

A variety of individuals were observed taking part in maternal and newborn care; we refer to these individuals as actors in our analysis. At the HCF, actors were categorised into five groups: mothers, fathers, HCW (doctors, nurses and midwives), cleaners (employed by the HCF), and *visitors* – all individuals not employed by the HCF and not the child’s mother or father. At the home, actors were categorised into three groups in analysis: mothers, fathers and non-parental caregivers. Non-parental caregivers included all other individuals who were observed engaging in the newborn caregiving activities at the home and included household members, relatives, and other non-family visitors. Our analysis explored the frequency of hand hygiene opportunities and hand hygiene actions by type of actor (mothers, fathers, HCW, visitors, cleaners and non-parental caregivers), by patient care setting (HCF and home) and type of care provided (maternal and newborn care).

Qualitative data was transcribed into Microsoft Word (Redmond, Washington) and analysed in Microsoft Word and Excel (Redmond, Washington). Findings from HCF staff interviews around IPC related practices have been previously reported [29]; qualitative data reported here focus on responses around discharge information and newborn care in the home environment.

Any self-reported practices by the mothers were compared against structured observations results. Interview and field note transcripts were coded by one author and independently reviewed by another [29]. Thematic analysis was deductive, based on the hand hygiene moments for community newborn care [31] specifically; during newborn handling - before carrying or after bottom cleaning following defecation, and cord care/contact.

## Results

### Participant information

A total of 39 mothers were recruited across the six facilities for at least one observation period; 31 mothers during labour and delivery, 31 mothers during post-natal period at the HCF, and 30 mothers at home. Eight mothers dropped out of the study after the post-natal care observations due to observation fatigue or non-consenting household members. An additional 7 participants were recruited for facility discharge and home observations.

Mothers had similar characteristics across the observations and interviews. All participating mothers reported they were married with a mean age of 30 (range: 19–39), had 2 previous births (range: 0–6) and spent an average of 35 min travelling to the health care facility (range: 5–120). Fathers were present in 26/31 post-natal care observations and in 28/30 home observations.

## Postnatal care

### Water, sanitation, and hygiene facilities

Functioning handwashing facilities with soap were available in 2 of the 6 post-natal care wards; however, no material for hand drying was present. There was no other provision for handwashing within any of the post-natal care wards e.g. alcohol-based hand rub.

### Hand hygiene opportunities and action

A total of 291 hand hygiene opportunities were observed during the post-natal period, 27% related to maternal care (79/291) and 73% related to newborn care (212/291) (Table 1).

**Table 1:** Observed hand hygiene opportunities and actions within post-natal care ward

	Hand Hygiene Opportunities n	Hand hygiene actions n (%)		
		*Adequate*^*a*^	*Inadequate*^*b*^	*No Action*
**All observations**				
Mothers	61	2 (3)	11 (18)	48 (79)
Fathers	37	0 (0)	0 (0)	37 (0)
Healthcare workers	84	1 (1)	12 (14)	71 (84)
Cleaners	6	0 (0)	0 (0)	6 (100)
Visitors	103	0 (0)	0 (0)	103 (100)
**Total**	**291**	**3 (1)**	**23 (8)**	**265 (91)**
**Maternal care**^**c**^				
Mothers	16	0 (0)	3 (19)	13 (81)
Fathers	0	0 (0)	0 (0)	0 (0)
Healthcare workers	57	1 (2)	6 (10)	50 (88)
Cleaners	1	0 (0)	0 (0)	1 (100)
Visitors	5	0 (0)	0 (0)	5 (100)
**Total**	**79**	**1 (1)**	**9 (12)**	**69 (87)**
**Newborn care**^**d**^				
Mothers	45	2 (4)	8 (18)	35 (78)
Fathers	37	0 (0)	0 (0)	37 (100)
Healthcare workers	27	0 (0)	6 (22)	21 (78)
Cleaners	5	0 (0)	0 (0)	5 (100)
Visitors	98	0 (0)	0 (0)	98 (100)
**Total**	**212**	**2 (1)**	**14 (7)**	**196 (92)**

^a^Adequate hand hygiene action includes washing hands with soap and washing hands with soap and wearing clean gloves for aseptic procedures

^b^Inadequate hand hygiene action includes rinsing hands without using soap or wearing gloves for aseptic procedures without handwashing with soap prior to donning gloves

^c^Maternal care includes contact by the healthcare workers, intramuscular injections, IV-related procedures, changing perineal pads, and emptying urine pan

^d^Newborn care includes direct cord contact via cord cleaning or cord inspection and newborn handling (changing newborn’s diapers, cleaning newborns bottom following defecation, picking up and putting newborn down, rubbing newborn’s body with body oils and powders, cleaning newborn’s eyes, changing newborn’s clothes, drying newborn with cloth, wiping newborn’s face)

Visitors accounted for 37% (103/291) of all observed hand hygiene opportunities. The majority (95%) of visitors’ hand hygiene opportunities were during newborn care activities. The remaining observed hand hygiene opportunities were among HCW (29%), mothers (21%), fathers (13%) and cleaners (2%).

Across all actors, no hand hygiene action was observed in relation to 91% (265/291) of hand hygiene opportunities. Half (13/26) of all observed hand hygiene actions were by HCW and the other half (13/26) by mothers. No hand hygiene actions were conducted by fathers, visitors, or cleaners. Only 3 of 26 hand hygiene actions observed were categorized as *adequate* (handwashing with soap and water) - once by a HCW prior to inspecting a mother’s perineal stitches and twice by mothers; prior to cord cleaning and prior to carrying the newborn. The remaining 23 hand hygiene actions were *inadequate*, and included HCWs wearing gloves without washing hands with soap prior to glove use (12/26) and mothers rinsing hands with water only (11/26). Among HCW, half (6/12) of inadequate hand hygiene actions were during maternal care - mostly prior to IV related procedures including cannula insertion, changing IV therapy bags and inspecting the IV cannula site. Of the 212 hand hygiene opportunities observed during newborn care, 8% (17/212) were related to cord contact and the rest (195/212), were during other contact with the newborn (Table 2).

**Table 2:** Observed hand hygiene opportunities and hand hygiene actions related to newborn care in post-natal care wards

	Hand hygiene opportunities n	Hand hygiene actions n (%)		
		*Adequate*^*a*^	*Inadequate*^*b*^	*No Action*
**All observations**				
Mothers	45	2 (4)	8 (18)	35 (78)
Fathers	37	0 (0)	0 (0)	37 (100)
Healthcare workers	27	0 (0)	6 (22)	21 (78)
Cleaners	5	0 (0)	0 (0)	5 (100)
Visitors	98	0 (0)	0 (0)	98 (100)
**Total**	**212**	**2 (1)**	**14 (7)**	**196 (92)**
**Cord contact**^**c**^				
Mothers	7	1 (14)	0 (0)	6 (86)
Fathers	0	0 (0)	0 (0)	0 (0)
Healthcare workers	7	0 (0)	4 (57)	3 (43)
Cleaners	1	0 (0)	0 (0)	1 (100)
Visitors	2	0 (0)	0 (0)	2 (100)
**Total**	**17**	**1 (6)**	**4 (24)**	**12 (70)**
**Other newborn care**^**d**^				
Mothers	38	1 (3)	8 (21)	29 (76)
Fathers	37	0 (0)	0 (0)	37 (100)
Healthcare workers	20	0 (0)	2 (10)	18 (90)
Cleaners	4	0 (0)	0 (0)	4 (100)
Visitors	96	0 (0)	0 (0)	96 (100)
**Total**	**195**	**1 (1)**	**10 (5)**	**184 (94)**

^a^Adequate hand hygiene action includes washing hands with soap and washing hands with soap and wearing clean gloves for aseptic procedures

^b^Inadequate hand hygiene action includes rinsing hands without using soap or wearing gloves for aseptic procedures without handwashing with soap prior to donning gloves

^c^Cord contact includes direct cord contact via cord cleaning or cord inspection

^d^Other newborn care includes newborn handling outside cord care (changing newborn’s diapers, cleaning newborns bottom following defecation, picking up and putting newborn down, rubbing newborn’s body with body oils and powders, cleaning newborn’s eyes, changing newborn’s clothes, drying newborn with cloth, wiping newborn’s face)

Cord contact in the post-natal care ward was made by multiple actors - mothers, HCWs, cleaners and visitors. The majority (13/17) of cord contact hand hygiene opportunities happened prior to cord cleaning and the rest (4/17) were during umbilical cord stump inspections. Across the 17 cord contact hand hygiene opportunities observed, hand hygiene actions were conducted 5 times, all of which were prior to cleaning of the newborn’s cord. Only 1/5 of hand hygiene actions was adequately performed.

## Discharge

The average length of stay after birth across all six facilities was 35 h (range: 7–96 h). Standardized discharge procedures were reported in all but one HCF. Key informants reported that discharge procedures included specific health information that should be provided to mothers at the time of discharge. However, 9/33 mothers did not receive any discharge advice at all from the midwives (Table 3).

**Table 3:** Observed discharge advice given to mothers

Topic	Advice	Primary (*N* = 10)	Secondary (*N* = 10)	Tertiary (*N* = 13)
Mother care	Do not insert anything into the vagina	2	5	8
	Take rest and sleep	5	8	8
	Wash perineum daily and after faecal excretion	5	6	8
	Change sanitary pads every 4 to 6 h	5	7	8
	Wash used pads or dispose of them safely	4	4	8
	Wash the body daily.	5	9	8
	Avoid sexual intercourse until the perineum heals	3	5	5
Newborn care	Wash hands before handling baby	3	6	8
	How to care for the cord	6	9	8
	Sleep under an insecticide treated net	6	6	8
	Exclusive breastfeeding	6	7	8
Other	Other advice	4	7	4
	No advice	4	0	5
Drugs	Pain relief	9	9	12
	Antiseptic	2	7	7
	Vitamin C	8	8	9
	Vitamin B complex	8	9	5

When given, discharge advice covered both maternal and newborn care. Information on hand hygiene, typically related to newborn care; specifically cord care, baby handling and breastfeeding. During observations, approximately half (17/33) of mothers received advice on washing their hands before handling the baby and 70% (23/33) received information on clean cord care, including washing hands before and after applying chlorohexidine, applying chlorohexidine exclusively on the cord, and placing the cord outside the diapers. However, in follow-up qualitative interviews, the only hand hygiene related discharge information that mothers could recall was involving breast feeding practices.

*… First and foremost, I was told that it is not good for a breastfeeding mother to keep long nails, that it harbours dirt and that it is dangerous to the baby. Secondly, I must wash my hands before I breastfeed the baby. In fact, I must make sure that the environment where the baby stays is very clean.* **– Mother, tertiary facility**

## Home observations

The average duration of home observations was 5.1 h (range: 2.4–6.8 h) with an average of 101 hand hygiene opportunities recorded per observation (range: 30–180). Home observations started an average of 4 h after discharge from the facility (range: 0–21). Three out of thirty women were observed for more than 6 hours post discharge.

### Water sanitation and hygiene facilities

Half (16/30) of households had access to a water source within the home, 7 had access to an on plot water source, and 7 used a public shared water source. The majority (27/30) of households had stored water within their household at the time of the observation, and all but one (29/30) had soap at the household. Of the 30 households visited, 21 had a private latrine, 8 had access to a latrine shared with other households, and 1 did not have access to a latrine. Two-thirds of households (20/30) had a handwashing facility within the compound, but only 9 households with a handwashing facility had soap or another cleansing agent present at the site. There was an average of 15 non-parental caregivers observed across household observations (range: 3–39). Non-parental caregivers included household members, visiting relatives, and other visitors.

### Hand hygiene: opportunities and action

All interviewed mothers knew both when and how hands should be washed in the home environment:

*Before carrying my baby, I wash my hand, before carrying her and anytime I go to the toilet, I wash my hand before carrying my baby, even if I go to the kitchen to cook, I wash my hand even if I just go to urinate I wash my hand before carrying my baby. –* **Mother, secondary facility**

*When I want to take care of the cord, I will wash my hand because I will be bathing the baby. I will wash my hand before I carry the baby for bathing.* – **Mother, primary facility**

However, this knowledge was not reflected in practice. In only 1% (5/459) of all hand hygiene opportunities observed in the home environment was hand hygiene performed adequately and hands rinsed at another 3% (12/459) of hand hygiene opportunities (Table 4). Mothers conducted the majority (15/17) of the hand hygiene actions.

**Table 4:** Observed hand hygiene opportunities and hand hygiene actions in the household

	Hand hygiene opportunities n	Hand hygiene actions n (%)		
		*Adequate*^*a*^	*Inadequate*^*b*^	*No Action*
**All observations**				
Mothers	154	4 (3)	11 (7)	139 (90)
Fathers	7	0 (0)	0 (0)	7 (100)
Non-parental caregivers	298	1 (0.5)	1 (0.5)	296 (99)
**Total**	**459**	**5 (1)**	**12 (3)**	**442 (96)**
**Cord contact**^**c**^				
Mothers	16	2 (13)	1 (6)	13 (81)
Fathers	0	0 (0)	0 (0)	0 (0)
Non-parental caregivers	13	0 (0)	0 (0)	13 (100)
**Total**	**29**	**2 (7)**	**1 (3)**	**26 (90)**
**Other newborn care**^**d**^				
Mothers	138	2 (2)	10 (7)	126 (91)
Fathers	7	0 (0)	0 (0)	7 (100)
Non-parental caregivers	285	1 (0.5)	1 (0.5)	283 (99)
**Total**	**430**	**3 (1)**	**11 (3)**	**416 (96)**

^a^Adequate hand hygiene action includes washing hands with soap and washing hands with soap and wearing clean gloves for aseptic procedures

^b^Inadequate hand hygiene action includes rinsing hands without using soap or wearing gloves for aseptic procedures without handwashing with soap prior to donning gloves

^c^Cord contact includes direct cord contact via cord cleaning or cord inspection

^d^Other newborn care includes newborn handling outside cord care (changing newborn’s diapers, cleaning newborns bottom following defecation, picking up and putting newborn down, rubbing newborn’s body with body oils and powders, cleaning newborn’s eyes, changing newborn’s clothes, drying newborn with cloth, wiping newborn’s face)

Cord contact accounted for 6% (29/459) of all hand hygiene opportunities; the majority (22/29) related to cord cleaning. Adequate hand hygiene was observed during 2 of 29 cord contact-related hand hygiene opportunities.

Non-maternal caregivers performed a variety of activities in the household, many of which put them at potential risk of transmitting infections to newborns during caregiving [see Additional file 1]. Over half of newborns (19/30) were bathed within the first 6 hours of their arrival to the home and bathing often involved multiple non-maternal caregivers. In one household, a newborn was bathed by 6 different non-maternal caregivers during the observation period. Following bathing, 8/19 newborns were rubbed with oils, in some cases mixed with different substances including; cassava flour mixed in red oil; black soap; palm kernel oil; shea butter; garlic and raw egg.

Despite the clear role of non-maternal caregivers in newborn care, mothers reported that asking any caregivers beyond fathers to wash hands was not feasible. Mothers noted that the caregivers would *‘not be happy’* or that they would *‘become angry’* if they were asked to wash their hands:

*Some visitors are in a haste, when they come they do not wash their hand, they carry their baby, after they go – [if asked to wash hands] they will become angry. -* **Mother secondary facility**

*I can’t tell visitors like that! -* **Mother, tertiary facility**

Some mothers, however described strategies for protecting their newborn, mostly by using the baby wrap as a physical barrier between the skin and the non-parental caregivers’ contaminated hands.

*If I ask them to wash their hands, I don’t know what they will feel! That is why I cover my baby with a towel before they carry my baby -* **Mother, primary facility**

*He is already dressed and covered with a towel so their hand will not touch the baby’s skin -* **Mother, primary facility**

## Discussion

Our mixed methods exploratory study describes hand hygiene practices in the post-natal care ward, facility discharge and the home environment across six healthcare facilities in Nigeria. Our findings show a low prevalence of hand hygiene practice during post-natal care and in the home environment in the immediate post-birth period. Our study also provides data on the wide range of individuals who are involved in both maternal and newborn care along this continuum, including healthcare workers, cleaners, visitors, fathers, mothers, and non-parental caregivers. Not only were hand hygiene actions rare during our observation period, similarly to other studies, hand hygiene actions were largely inadequate; for example, HCW using gloves without having washed their hands with soap before [28, 32] and mothers and other caregivers rinsing hands with water only [21, 22, 33, 34]. Visitors in the health facility and non-maternal caregivers at the home accounted for the majority of observed hand hygiene opportunities, particularly around newborn care, but no hand hygiene actions were observed by these groups.

Handwashing with soap promotion will fail if inadequate infrastructure is in place. Unlike the labour and delivery rooms for facilities included in this study [29] the vast majority of post-natal care wards lacked adequate hand hygiene infrastructure and/or supplies. The lack of functioning hygiene infrastructure and supplies is commonly reported as a major barrier in both HCFs and at home to practicing hygienic behaviours [35–37]. The provision of handwashing facilities with soap at all points of care are the basic requirements for HCFs according to global monitoring strategies [38]. Point of care can be recognised as the place where the patient, the HCW, and the provision of care or treatment come together [12]. Our study shows that in the context of newborn care in the HCF, the ‘point of care’ should expand beyond delivery ward and include post-natal care areas. In the absence of hygiene infrastructure, alcohol-based hand rubs have been shown to improve hand hygiene practices and may be an effective low cost intervention for consideration [39–43].

The prevalence of appropriate hand hygiene by HCW during labour and delivery has been found to be generally low [17, 28, 32, 44]. This study finds that HCW maintain inadequate hand hygiene practices into the post-natal care period. Increased emphasis on HCW washing hands with soap and appropriate glove use in post-natal care is needed and should be incorporated into standard quality of care and IPC improvement programs. Previous data from participating facilities shows that current models of step-down training on hand hygiene and IPC are inadequate, didactic, irregularly given and accompanied by little to no oversight [29]. In addition to general improvements to the overall infection control and hand hygiene training [41, 45–47], our data suggest that adherence to hand hygiene protocols specific to the post-natal care areas should be emphasised and integrated into multi-modal infection control strategies [41, 47].

The discharge process presents a valuable but under-utilised opportunity to promote hand hygiene among all caregivers along the care continuum from facility to the home. Another study in Edo state, Nigeria found that mothers who practiced hygienic cord care reported that nurses had a stronger influence on mothers’ behaviours compared to other caregivers [48]. Together with standardised discharge protocols and checklists [49], additional moments in the post-natal ward need to be identified to enable HCWs to provide and reinforce accurate, standardised, and simplified information in a way that it can be remembered and practiced by all caregivers while in the post-natal ward and at home.

Our observational study demonstrates the important role that non-maternal caregivers play during care both in the post-natal care ward and in the home environment. Other facility based studies in LMIC have documented the integral role of family members in patient management, their accompanying hand hygiene practices and the potential exposure risk they carry. For example, studies in Bangladesh reported that compliance of family members providing inpatient care ranged between 0% [50] and 3% [34]. Studies on hygiene during neonatal care in the home environment focus primarily on the new mothers or birth attendants [18, 23, 24, 51]. Non-maternal caregivers are not only actively engaged in newborn care in these settings, but they are also important drivers of the mothers’ handwashing behaviours [21, 22]. Interventions may potentially overlook the critical role and engagement of fathers and extended family members in newborn contact [52]. In a tertiary hospital in India, Biswal et al. [53] reported a 13% improvement of family member compliance following the implementation of a hand hygiene improvement strategy that included a caregiver-specific training component. Understanding the drivers of behaviours of the wider context within which the mother exists and how these behaviours are informed and modified by both the physical and social environment can help in the development of new interventions that target wider audiences in both the home and the healthcare [50, 52, 54].

The small number of facilities for this observational study limit the generalisability of our findings to beyond these study sites. Our study had a participant dropout rate of 26% prior to the home observations, which may have introduced bias into our study if the participants who dropped out systematically differed from those who remained or were later recruited into the study. However, data suggests that drop-outs and new enrolments were similar in age, previous births, and time spent travelling to clinic. Reactivity by participants to the presence of an observer may have led some actors to increase hand hygiene compliance [55]. However, this reactivity was minimised by avoiding any explicit mention of handwashing behaviour being the aim of the study and carrying out the observations before the household interviews and overall low levels of hand hygiene compliance observed in this study suggest that the impact of reactivity on handwashing behaviours was likely minimal.

## Conclusion

Our study shows that hand hygiene along the entire continuum of maternal and newborn care is inadequate. In addition to the delivery room, future behaviour change interventions need to address hand hygiene practices within the post-natal care ward and early days at home and target a wider range of caregivers than mothers and healthcare workers. More in-depth research is required to understand the drivers of hand hygiene practices for all actors involved in maternal and newborn care in the immediate post-birth period and targeted interventions needed to improve hand hygiene practices developed. However, the basic provision of appropriate hygiene infrastructure in post-natal care wards is an urgent action that should prioritized as part of global efforts to expand water, sanitation, and hygiene coverage in healthcare facilities.

## Supplementary information

**Additional file 1.** Activities performed by non-maternal caregivers

## Data Availability

The datasets used and/or analysed during this study are available from the corresponding author on reasonable request.

## Abbreviations

HCAI: Healthcare associated infections
HCF: Healthcare facilities
HCW: Healthcare workers
IPC: Infection prevention and control
IV: Intravenous
LMIC: Low- and middle-income countries
WHO: World Health Organisation
