# Supplementary material for: Hygiene along the continuum of care in the early post-natal period: an observational study in Nigeria
Source: BMC Pregnancy Childbirth. 2020 Oct 6;20:589. doi: 10.1186/s12884-020-03282-3 (PMC7541299; doi:10.1186/s12884-020-03282-3)
Supplement: Supplementary file 1 — Additional file 1. Activities performed by non-maternal caregivers [file 12884_2020_3282_MOESM1_ESM.docx]

**Additional File 1: Activities performed by non-maternal caregivers**

| **Activities performed by non-paternal caregivers** |
| --- |
| Food preparation for visitors and other household members |
| Caretaking of other children in the household e.g. feeding, bathing, playing |
| General house cleaning activities |
| Cord care and inspection |
| Bathing the newborn including drying and dressing activities |
| Rubbing newborn’s body with oil, powder, butter following bathing |
| Cleaning the newborn’s bottom following defecation |
| Changing newborns diapers/diapers following defecation |
| Moving newborn to and from bed or mothers’ arms |
| Cleaning newborn’s eyes |
| Visiting the toilet |
| Caretaking of the mother e.g. assisting mother to go to the toilet, giving medication |
| Leading religious activities |
| Welcoming and entertaining visitors |
